# Supplementary material for: Predicting gene expression changes upon epigenomic drug treatment
Source: F1000Res. 2025 May 2;12:1089. Originally published 2023 Sep 1. [Version 3] doi: 10.12688/f1000research.140273.3 (PMC12103705; doi:10.12688/f1000research.140273.3)
Supplement: Peer review history

# Reviewer report: Reviewer response for version 3

**DOI:** 10.5256/f1000research.181146.r383150

**Published:** 2025-05-24

**Authors:** Ashwini Kumar

**Affiliations:** 1 Sharda University, Greater Noida, Uttar Pradesh, India

The article titled "Predicting gene expression changes upon epigenomic drug treatment" is an interesting approach to show the effect of drug treatment on the genomic and epigenomic changes in cancer cells and prepare a model that can predict the post-treatment changes across the cancer types. Though drugs that target the epigenomic alterations in cancer have been clinically used, however they lack locus-specific action that may result in unpredictable adverse effects. Thus. the approach presented in this manuscript tackles pre- and post-treatment gene expression and pre-treatment epigenomic profile, and paves a way to predict the locus-specific effect(s) of the drugs that may lead us to personalize the therapy, and also lead us to develop more rational drugs.

 Although such models can be made on in vitro studies involving various cancer cells, however this manuscript presents a potential new approach to tackle the issue of epigenomic changes that have substantial effect pre- and post-treatment. These models can help the researchers develop better drug development strategies while clinicians to have better therapeutic strategies minimizing the adverse effects.

Is the work clearly and accurately presented and does it cite the current literature?

Yes

If applicable, is the statistical analysis and its interpretation appropriate?

Not applicable

Are all the source data underlying the results available to ensure full reproducibility?

Yes

Is the study design appropriate and is the work technically sound?

Yes

Are the conclusions drawn adequately supported by the results?

Yes

Are sufficient details of methods and analysis provided to allow replication by others?

Yes

Reviewer Expertise:

Drug delivery and drug discovery

I confirm that I have read this submission and believe that I have an appropriate level of expertise to confirm that it is of an acceptable scientific standard.

# Reviewer report: Reviewer response for version 3

**DOI:** 10.5256/f1000research.181146.r383152

**Published:** 2025-05-24

**Authors:** Vivek Dhar Dwivedi

**Affiliations:** 1 Quanta Calculus, Greater Noida, India

The current study proposes a novel and interesting strategy to predict gene expression changes using histone modification patterns, with a focus on H3K27 acetylation, in response to drug exposure. The findings may play a key role in enhancing personalized treatment strategies for cancer patients undergoing histone deacetylase inhibitor therapy.

 Still, a few minor but important concerns need to be addressed prior to its acceptance.

 1. Authors should explain why they use only the mentioned 2 cell lines in the current study.

 2. Please provide an explanation if this kind of approach will also work for other histone marks?

 3. Reason behind selecting the SVM method in the case of Figure 5.

Is the work clearly and accurately presented and does it cite the current literature?

Yes

If applicable, is the statistical analysis and its interpretation appropriate?

Not applicable

Are all the source data underlying the results available to ensure full reproducibility?

Yes

Is the study design appropriate and is the work technically sound?

Yes

Are the conclusions drawn adequately supported by the results?

Yes

Are sufficient details of methods and analysis provided to allow replication by others?

Yes

Reviewer Expertise:

Bioinformatics

I confirm that I have read this submission and believe that I have an appropriate level of expertise to confirm that it is of an acceptable scientific standard.

# Reviewer report: Reviewer response for version 3

**DOI:** 10.5256/f1000research.181146.r382129

**Published:** 2025-05-24

**Authors:** Sukhen Das Mandal

**Affiliations:** 1 Ghani Khan Choudhary Institute of Engineering and Technology, Malda, West Bengal, India

All my concerns have been addressed satisfactorily. Therefore, this article may be approved.

Is the work clearly and accurately presented and does it cite the current literature?

Partly

If applicable, is the statistical analysis and its interpretation appropriate?

Not applicable

Are all the source data underlying the results available to ensure full reproducibility?

No

Is the study design appropriate and is the work technically sound?

Partly

Are the conclusions drawn adequately supported by the results?

No

Are sufficient details of methods and analysis provided to allow replication by others?

Yes

Reviewer Expertise:

RNA biology, Machine learning

I confirm that I have read this submission and believe that I have an appropriate level of expertise to confirm that it is of an acceptable scientific standard.

# Reviewer report: Reviewer response for version 2

**DOI:** 10.5256/f1000research.159982.r267047

**Published:** 2025-05-07

**Authors:** Indrakant K. Singh

**Affiliations:** 1 Deshbandhu College, University of Delhi, Delhi, India

The study presents a novel machine learning approach to predict gene expression changes from histone modification data, specifically H3K27 acetylation, after drug treatment. This could have significant implications for personalized cancer treatment with histone deacetylase inhibitors.

 However, there are some concerns that need to be addressed before accepting.

 1. The rationale behind choosing different parameters (e.g., z-scores of normalized read counts in Figure 6 versus normalized read counts in Figure 3) is not adequately explained. Consistency in data presentation or a clear justification for differences is needed.

 2.     The manuscript needs more detailed descriptions of certain methods. For example, the process of peak calling in ChIP-seq analysis and the significance of the log10 fold change in differential expression analysis are not clearly described. Detailed methodological transparency is essential for replication and understanding

 3.     Some results and their descriptions appear contradictory or unclear. For instance, the authors state that HCT116 genes showed higher concentrations of H3K27Ac marks, but this is not consistent with what is shown in Figure 6. Such inconsistencies need to be addressed and clarified.

 I recommend minor revision.

Is the work clearly and accurately presented and does it cite the current literature?

Partly

If applicable, is the statistical analysis and its interpretation appropriate?

Partly

Are all the source data underlying the results available to ensure full reproducibility?

Yes

Is the study design appropriate and is the work technically sound?

Yes

Are the conclusions drawn adequately supported by the results?

Yes

Are sufficient details of methods and analysis provided to allow replication by others?

Yes

Reviewer Expertise:

Bioinformatics and Cancer Biology

I confirm that I have read this submission and believe that I have an appropriate level of expertise to confirm that it is of an acceptable scientific standard.

# Reviewer report: Reviewer response for version 2

**DOI:** 10.5256/f1000research.159982.r243027

**Published:** 2024-08-01

**Authors:** Sukhen Das Mandal

**Affiliations:** 1 Ghani Khan Choudhary Institute of Engineering and Technology, Malda, West Bengal, India

In the paper authors developed machine learning models to predict gene expression changes for HDACi treatment. Though it will enrich current understanding about how epigenomic drugs can change expression of genes, there are multiple/some issues those need to be addressed. Suggestions:

 1. GO analysis of upregulated and down regulated genes for HDACi treatment showed that genes are associated with different types of GO terms for different cell lines. Whether it is because different cell lines respond differently to HDACi or different types of HDACi act through different mechanisms, you should discuss it more elaborately in the result section. 

 2. H3K27Ac profile for treated vs untreated should be checked for up and down regulated genes. This can verify whether change of H3K27Ac causes transcriptional change which leads to expression change or post transcriptional regulation like RNA stability causes the change of the expression. 

 3. "(3) this trend was also true in gene body but only at mid and higher dosage" or " (5) RH4 trends at 1 μm dose of Entinostat most resembled the patterns at 75 nM dose of largazole in HCT116" -  These statements do not match with the figures. Kindly explain these more clearly. 

 4. The length of GB is different for every gene. The bin size of the genes having larger GB size is higher compared to genes having shorter GB. As a longer bin having more numbers of nucleotides gives the possibility of higher numbers of H3K27Ac count. So, authors should consider the length of GB into their analysis and should check whether the pattern persists after normalisation by the length of the GB of a gene.

 5. "As shown below, with few exceptions, for the first gene set, HCT116 genes showed higher concentration of H3K27Ac marks and for the second gene set, the opposite was true (Figure 6B)". Again these statements are not matching with the figures rather the opposite may be true or there is no clear pattern to mention. Kindly recheck your results and explain it properly. 

 6. The reference of the figure 5A is not there in the text.

Is the work clearly and accurately presented and does it cite the current literature?

Partly

If applicable, is the statistical analysis and its interpretation appropriate?

Not applicable

Are all the source data underlying the results available to ensure full reproducibility?

No

Is the study design appropriate and is the work technically sound?

Partly

Are the conclusions drawn adequately supported by the results?

No

Are sufficient details of methods and analysis provided to allow replication by others?

Yes

Reviewer Expertise:

RNA biology, Machine learning

I confirm that I have read this submission and believe that I have an appropriate level of expertise to confirm that it is of an acceptable scientific standard, however I have significant reservations, as outlined above.

# Response

**Published:** 2025-04-25

**Authors:** Piyush Agrawal

**Affiliations:** Division of Medical Research, SRM Institute of Science and Technology (Deemed to be University), Kattankulathur, Tamil Nadu, India

**Reviewer 3**

 In the paper authors developed machine learning models to predict gene expression changes for HDACi treatment. Though it will enrich current understanding about how epigenomic drugs can change expression of genes, there are multiple/some issues those need to be addressed.

**Suggestions:**

 1. GO analysis of upregulated and downregulated genes for HDACi treatment showed that genes are associated with different types of GO terms for different cell lines. Whether it is because different cell lines respond differently to HDACi or different types of HDACi act through different mechanisms, you should discuss it more elaborately in the result section.

**Response:** We thank the reviewer for this insightful comment. In response, we have expanded the discussion in the Results section to clarify the biological relevance of the distinct GO term enrichments observed across different cell lines following HDACi treatment.

 Specifically, we observed that **upregulated genes were consistently enriched for developmental and cell signaling processes**, while **downregulated genes were predominantly associated with cell cycle-related functions**. This pattern suggests that HDAC inhibition may generally suppress proliferation while activating differentiation or lineage-specifying pathways. However, the specific GO terms varied somewhat across different cell lines, which may either reflect context-specific effects of HDACi, or the **differences in the specific HDAC inhibitors used**; however, since different HDACi compounds were used in different cell lines, it is not possible to distinguish between these two possibilities.

 We have now added this interpretation to the Results section (page 5, lines 139–149).

 The following section has been added in the revised manuscript:

*“Gene Ontology (GO) enrichment analysis revealed that genes **upregulated by HDACi treatment** were broadly involved in **developmental processes and cell signaling pathways**, suggesting a potential shift toward differentiation or activation of lineage-specific programs. In contrast, **downregulated genes were strongly enriched for cell cycle-related processes**, consistent with the known anti-proliferative effects of HDAC inhibitors. Notably, while this overall pattern was observed across all cell lines, the specific GO terms and pathways varied between them. This indicates that **cell line-specific factors—such as differences in chromatin accessibility, baseline gene expression, or mutational background—may influence the transcriptional response to HDACi**. Some variability may also stem from the **use of different HDAC inhibitors**, which may target different HDAC isoforms and thus modulate distinct regulatory networks. Disentangling thse two possibilities however will necessitate a broader set of experiments involving multiple cell lines and multiple HDACi with proper experimental design.”*

 2. H3K27Ac profile for treated vs untreated should be checked for up and down regulated genes. This can verify whether change of H3K27Ac causes transcriptional change which leads to expression change or post transcriptional regulation like RNA stability causes the change of the expression.

**Response:** We completely agree and had considered this. Unfortunately, however the post-treatment H3K27Ac data is currently not available.

 3. "(3) this trend was also true in gene body but only at mid and higher dosage" or " (5) RH4 trends at 1 μm dose of Entinostat most resembled the patterns at 75 nM dose of largazole in HCT116" - These statements do not match with the figures. Kindly explain these more clearly.

**Response:** We regret the lack of clarity. For point 3, we are talking about higher distribution of H3K27Ac epigenomic marks in gene body regions of the downregulated genes for HCT cell line with concentration of 75nM and 300nM. In the figure, in case of 75nM, gene body bin 1-3, 6 and 8-10 shows higher H3K27Ac read density concentration, where in remaining bins its nearly equal among up and downregulated genes. Likewise, in case of 300nM, majority of the gene body shows higher H3K27Ac distribution then promoter region except bin 1, and 10. In revised manuscript, we have now revised the statement for clarity.

 4. The length of GB is different for every gene. The bin size of the genes having larger GB size is higher compared to genes having shorter GB. As a longer bin having more numbers of nucleotides gives the possibility of higher numbers of H3K27Ac count. So, authors should consider the length of GB into their analysis and should check whether the pattern persists after normalisation by the length of the GB of a gene.

**Response: **We appreciate this. However, we would like to clarify that **our analysis already incorporates normalization that accounts for these differences**.

 Specifically, as mentioned in the Methods section, **we normalized the read counts within each genomic bin to TPM (Transcripts Per Million)** using the **Rsubread** package. TPM normalization adjusts for both sequencing depth and **bin length**, thereby mitigating the potential bias introduced by longer bins containing more nucleotides.

 Thus, the enrichment patterns we observed are not confounded by gene length variation and reflect true biological signal distribution rather than technical artifacts. We have now made this point more explicit in the manuscript for clarity.

 5. "As shown below, with few exceptions, for the first gene set, HCT116 genes showed higher concentration of H3K27Ac marks and for the second gene set, the opposite was true (Figure 6B)". Again, these statements are not matching with the figures rather the opposite may be true or there is no clear pattern to mention. Kindly recheck your results and explain it properly.

**Response:** We thank the reviewer for pointing out this mistake and we also regret the misinterpretation of the results. In the revised manuscript, we have clarified this for the better explanation. **Page 7; Lines 217:220.**

 “As shown below, we do not observe any clear pattern for the H3K27Ac read density among the two cell lines, except at TSS and gene body 1 region, where we observed lower H3K27Ac read distribution for the upregulated genes **(Figure 6A&B)”**.

 6. The reference of the figure 5A is not there in the text.

**Response:** Figure 5A is referenced on Page No. 7, Line No 201.

# Reviewer report: Reviewer response for version 2

**DOI:** 10.5256/f1000research.159982.r239600

**Published:** 2024-02-22

**Authors:** Daiqing Liao, Zhiguang Huo

**Affiliations:** 1 Department of Anatomy and Cell Biology, College of Medicine & UF Health Cancer Center, University of Florida, Gainesville, USA; 2 Department of Biostatistics, University of Florida, Gainesville, Florida, USA

Cancer epigenome underpins oncogenic gene expression. Histone acetylation is a major epigenomic event that impacts the transcriptome. The dynamic action of histone acetyltransferases (HATs) and deacetylases (HDACs) shape the acetylome. Small molecule inhibitors of both HATs and HDACs are promising agents for cancer therapy. Several HDAC inhibitors (HDACi) have been approved for treating cutaneous T-cell lymphoma and peripheral T-cell lymphoma. HDACi profoundly perturbs the transcriptome ^1^. Thousands of genes can be downregulated and upregulated after treatment with an HDACi ^2,3^. Pretreatment epigenome could influence transcriptomic perturbation by HDACi, although this has not been thoroughly examined. The manuscript titled “Predicting gene expression changes upon epigenomic drug treatment” by Agrawal, Gopalan, and Hannenhalli assessed whether the pretreatment epigenomic profile of cells could predict gene expression alterations induced by HDACi. The authors used the levels of a single histone mark, namely H3K27ac, in divided bins (21 genomic bins in total) from 2 kb upstream of the transcription start site (TSS) to the end of gene body and data of differentially expressed genes (DEGs) as inputs to study the relationship between the pretreatment H3K27ac levels and the transcription outputs in several machine learning models. The authors found that the pretreatment H3K27ac patterns could predict the post-treatment up versus downregulated genes with reasonable accuracy. Essentially, gene expression levels due to HDACi treatment seem to be inversely correlated with the levels of H3K27ac before treatment, which is observed in two cell lines treated with two HDACi of different chemical classes (entinostat and largazole). While the results are interesting, several significant issues should be addressed.

  

1. The authors should also generate the feature importance score, e.g., the importance score for the random forest model (all gene models). This would be informative for readers to understand which genomic bin is most important to predict gene expression regulation.
2. The authors should show the p-value for each comparison pair in Figure 3.
3. The authors’ description “with few exceptions, for the first gene set, HCT116 genes showed higher concentration of H3K27Ac marks, and for the second gene set, the opposite was true (Figure 6B), consistent with the patterns in Figure 3” (page 9) seems contradictory to what is shown in Figure 6. This must be clarified.
4. In Figure 6, the z-scores of normalized read counts are used, while normalized read counts are shown in Figure 3. What is the rationale for using different parameters? Also, the p-value for each pair comparison should be shown in Figure 6.
5. Although the authors acknowledged that different epigenomic marks should be included in future studies, they are encouraged to analyze at least whether a different histone mark associated with active transcription, such as H3K4me3, would exhibit a similar predictive power as H3K27ac.
6. Another major limitation of the manuscript is that data based on only two cell lines and two HDACi are analyzed. An expanded study of data from a diverse set of samples (cells and tumors) treated with additional HDACi should improve the validity and rigor of the study. 

Is the work clearly and accurately presented and does it cite the current literature?

Partly

If applicable, is the statistical analysis and its interpretation appropriate?

Partly

Are all the source data underlying the results available to ensure full reproducibility?

Yes

Is the study design appropriate and is the work technically sound?

Yes

Are the conclusions drawn adequately supported by the results?

Partly

Are sufficient details of methods and analysis provided to allow replication by others?

Yes

Reviewer Expertise:

bioinformatics (Zhiguang Huo) and epigenetics (Daiqing Liao)

We confirm that we have read this submission and believe that we have an appropriate level of expertise to state that we do not consider it to be of an acceptable scientific standard, for reasons outlined above.

## References

[1] : Chemical genomics reveals histone deacetylases are required for core regulatory transcription. *Nat Commun* .2019;10(1) : 10.1038/s41467-019-11046-7 3004 10.1038/s41467-019-11046-7 31285436 PMC6614369

[2] : Histone deacetylase (HDAC) inhibitor kinetic rate constants correlate with cellular histone acetylation but not transcription and cell viability. *J Biol Chem* .2013;288(37) : 10.1074/jbc.M113.490706 26926-43 10.1074/jbc.M113.490706 23897821 PMC3772242

[3] : HDAC3 and HDAC8 PROTAC dual degrader reveals roles of histone acetylation in gene regulation. *Cell Chem Biol* .2023;30(11) : 10.1016/j.chembiol.2023.07.010 1421-1435.e12 10.1016/j.chembiol.2023.07.010 37572669 PMC10802846

# Reviewer report: Reviewer response for version 1

**DOI:** 10.5256/f1000research.153610.r203232

**Published:** 2023-09-20

**Authors:** Angelika Merkel

**Affiliations:** 1 Bioinformatics Unit, Josep Carreras Leukemia Research Institute (IJC), Barcelona, Spain

Agrawal et al have presented a comparison of machine learning approaches to predict gene expression from the distribution of histone marks, specifically H3K27 acetylation.

 Albeit interesting and potentially very useful for cancer specific and personalized treatment with histone deacetylase inhibitors as the authors suggest, the latter has actually not been addressed. The authors present a proof-of-concept study for predicting gene expression from histone marks but not do not address variations in tissues, individual or disease type.

 As such, this is not a novel approach as there have been numerous studies before addressing the prediction of gene expression (e.g. Karlic et 2010 ^1^, Singh et al 2016 ^2^, Chen et al 2022 ^3^). The authors have failed to cite any of the current literature on the topic.

 Methods and results are presented in a somewhat rough manner and need improvement:

- Abstract and introduction contain poor grammar and sentence structure ('genomic specificity" is not a new term to be defined, better to to use target specificity)
- Why were the HCT166 and RH4 cell lines chosen and what are these exactly (need description)?
- Was peak calling performed to eliminate background noise in the Chip-seq analysis? 
- Was differential expression analysis performed (normalization, statistical analysis, etc.)? What is the significance of the log10fold change? 
- The GO analysis is repeated in the methods and results section 
- Figure 3: The scale among plots differ; gene bins are of variable size and larger promoter bins. Why have they been plotted them together?

Is the work clearly and accurately presented and does it cite the current literature?

No

If applicable, is the statistical analysis and its interpretation appropriate?

Partly

Are all the source data underlying the results available to ensure full reproducibility?

Yes

Is the study design appropriate and is the work technically sound?

Partly

Are the conclusions drawn adequately supported by the results?

Partly

Are sufficient details of methods and analysis provided to allow replication by others?

Partly

Reviewer Expertise:

transcriptomics, epigenetics, bioinformatics

I confirm that I have read this submission and believe that I have an appropriate level of expertise to state that I do not consider it to be of an acceptable scientific standard, for reasons outlined above.

## References

[1] : Histone modification levels are predictive for gene expression. *Proc Natl Acad Sci U S A* .2010;107(7) : 10.1073/pnas.0909344107 2926-31 10.1073/pnas.0909344107 20133639 PMC2814872

[2] : DeepChrome: deep-learning for predicting gene expression from histone modifications. *Bioinformatics* .2016;32(17) : 10.1093/bioinformatics/btw427 i639-i648 10.1093/bioinformatics/btw427 27587684

[3] : Predicting gene expression from histone modifications with self-attention based neural networks and transfer learning. *Front Genet* .2022;13: 10.3389/fgene.2022.1081842 1081842 10.3389/fgene.2022.1081842 36588793 PMC9797047

# Response

**Published:** 2023-12-09

**Authors:** Piyush Agrawal

**Affiliations:** Division of Medical Research, SRM Institute of Science and Technology (Deemed to be University), Kattankulathur, Tamil Nadu, India

Agrawal et al have presented a comparison of machine learning approaches to predict gene expression from the distribution of histone marks, specifically H3K27 acetylation.

 Albeit interesting and potentially very useful for cancer specific and personalized treatment with histone deacetylase inhibitors as the authors suggest, the latter has actually not been addressed. The authors present a proof-of-concept study for predicting gene expression from histone marks but not do not address variations in tissues, individual or disease type.

**Response: **We thank the reviewers for finding our work and interesting and potentially useful. Although we agree with reviewer regarding the importance of addressing variations in tissues, individual or disease type, unfortunately, lack of appropriate data currently prohibits such analysis. Besides the 2 cell lines used in this study, we didn’t come across any dataset (clinical/cell line) where for a given patient, both epigenomic marks and gene expression data is provided for both pre and post drug treatment. We would be happy to do the additional analysis if the reviewer can kindly point us to such data.

 As such, this is not a novel approach as there have been numerous studies before addressing the prediction of gene expression (e.g. Karlic et 2010 ^1^ , Singh et al 2016 ^2^ , Chen et al 2022 ^3^ ). The authors have failed to cite any of the current literature on the topic.

**Response:** We thank the reviewer for pointing out these studies, however, we want to clarify that the above-mentioned studies address a different question than ours, which is “to predict gene expression from the epigenome in a specific context”. Whereas we are interested in predicting the effect on the expression upon drug treatment, given the epigenomic profile in the pre-treatment sample. Our question necessitates availability of pre- and post-treatment gene expression and pre-treatment epigenomic profile, while these previous approaches are not concerned with the changes upon drug treatment. In the revised manuscript, we have highlighted this difference in the Introduction.

 Methods and results are presented in a somewhat rough manner and need improvement:

 Abstract and introduction contain poor grammar and sentence structure ('genomic specificity" is not a new term to be defined, better to to use target specificity)

**Response:**

****

 Why were the HCT166 and RH4 cell lines chosen and what are these exactly (need description)?

**Response: **We regret lack of clarity. HCT116 and RH4 cell lines were chosen because the data we required for our study was available only for these 2 cell lines, namely, pre-treatment epigenomic profile and pre- and post-treatment gene expression data. HCT116 is human colorectal carcinoma cell line initiated from an adult male whereas RH4 cell line is for studying alveolar rhabdomyosarcoma and is belongs to soft tissue lineage. In the revised manuscript, we have added the description of these cell lines in the Methods section.

 Was peak calling performed to eliminate background noise in the Chip-seq analysis? 

**Response:** No, peak calling wasn’t performed in the current study. The goal of our analysis was to compare changes in H3K27ac read density upon HDAC treatment. Peak calling represents a 0/1 binarization of a continuous variable (in this case, normalized read counts in promoters and gene bodies). Our model uses the epigenomic signal intensity in specific windows around the gene magnitude, making peak-calling unnecessary.  

 Was differential expression analysis performed (normalization, statistical analysis, etc.)? What is the significance of the log10fold change? 

**Response: **The mean Log-TPM values of each gene was computed for both treated and untreated conditions, with the Log-FC computed as the difference between treated and untreated conditions. The top 1000 most up-regulated and 1000 most down-regulated genes were considered to be differentially expressed. We mistakenly mentioned Log10, which is now corrected to Log2. We have corrected this in the revised version **.**

 The GO analysis is repeated in the methods and results section 

**Response:** We want to clarify that in Methods Section, we mentioned the software and command only used for GO analysis, however, in Result section, we discussed the enriched terms associated with the up and downregulated genes.

 Figure 3: The scale among plots differ; gene bins are of variable size and larger promoter bins. Why have they been plotted them together?

**Response:** We agree with the reviewer that scale among plots differ and gene bins are of variable size. The only reason for plotting them together was to analyze all the 21 features together in single image i.e. change in distribution of the pre-treatment epigenomic marks in up and down genes post treatment.

**Is the work clearly and accurately presented and does it cite the current literature?**

 No

**Is the study design appropriate and is the work technically sound?**

 Partly

**Are sufficient details of methods and analysis provided to allow replication by others?**

 Partly

**If applicable, is the statistical analysis and its interpretation appropriate?**

 Partly

**Are all the source data underlying the results available to ensure full reproducibility?**

 Yes

**Are the conclusions drawn adequately supported by the results?**

 Partly

**References**

 1. Karlić R, Chung HR, Lasserre J, Vlahovicek K, et al.: Histone modification levels are predictive for gene expression. *Proc Natl Acad Sci U S A*. 2010; **107** (7): 2926-31 PubMed Abstract | Publisher Full Text

 2. Singh R, Lanchantin J, Robins G, Qi Y: DeepChrome: deep-learning for predicting gene expression from histone modifications. *Bioinformatics*. 2016; **32** (17): i639-i648 PubMed Abstract | Publisher Full Text

 3. Chen Y, Xie M, Wen J: Predicting gene expression from histone modifications with self-attention based neural networks and transfer learning. *Front Genet*. 2022; **13**: 1081842 PubMed Abstract | Publisher Full Text
